# Association between the vaginal and uterine microbiota and the risk of early embryonic arrest

**DOI:** 10.3389/fmicb.2023.1137869

**Published:** 2023-03-22

**Authors:** Li Wang, Junyu Chen, Lin He, Hanbo Liu, Yan Liu, Zonghui Luan, Hong Li, Weixin Liu, Mengjun Luo

**Affiliations:** ^1^Department of Clinical Laboratory, School of Medicine, Chengdu Women’s and Children’s Central Hospital, University of Electronic Science and Technology of China, Chengdu, Sichuan, China; ^2^Department of Gynecology, School of Medicine, Chengdu Women’s and Children’s Central Hospital, University of Electronic Science and Technology of China, Chengdu, Sichuan, China; ^3^Key Laboratory of Reproductive Medicine, Sichuan Provincial Maternity and Child Health Care Hospital, The Affiliated Women’s and Children’s Hospital of Chengdu Medical College, Chengdu, Sichuan, China

**Keywords:** embryo arrest, microbiome, pregnancy, vagina, uterus

## Abstract

The aim of this study was to explore the microecological distribution and differences in the uterus and vaginal microbiome in women with early embryonic arrest and those with normal pregnancy by high-throughput sequencing. We systematically sampled the vaginal and uterine microbiomes of 56 pregnant women, namely, 38 patients with early embryonic arrest and 18 pregnant women with normal pregnancy-induced abortion. We obtained colonization data by 16S rRNA gene amplicon sequencing. In the vagina, *Lactobacillus*, *Bacteroidetes* and *Helicobacter* exhibited significant differences between the groups. We further found that *Lactobacillus iners*, *Lactobacillus crispatus*, *Lactobacillus gasseri* and *Lactobacillus jensenii* were the most dominant *Lactobacillus* species and that *L. iners* was significantly different between the groups. Receiver operating characteristic (ROC) curve analysis confirmed that *Ensifer* had the highest predictive value for early embryonic arrest. In the uterine cavity, we determined that Proteobacteria, Bacteroidetes, Firmicutes and Actinobacteria were the dominant bacteria at the phylum level and that *Bacteroides*, *Pseudarthrobacter*, *Lactobacillus* and *Ralstonia* were the dominant genera. Further classification of *Lactobacillus* revealed that *L. iners*, *L. crispatus*, *L. gasseri*, and *L. jensenii* were the main species. There was a significant difference in *L. jensenii* between the normal pregnancy group and early embryonic arrest group. Random forest analysis revealed 18 different genera in the uterus, and ROC curve analysis indicated that Candidatus Symbiobacter, Odoribacter, Blautia, Nocardioides and Ileibacterium had a certain predictive value.

## Introduction

Early embryonic arrest is defined as the ultrasound diagnostic of an average diameter of the gestational sac diameter larger than 25 mm, no heartbeat, a crown-rump length exceeding 7 mm, and no fatal cardiac activity by the original ultrasound examination (Hendriks and MacKenzie, 2019). Early embryonic arrest is a component of early spontaneous abortion and a common complication in early pregnancy that can lead to adverse pregnancy outcomes. At present, the main treatment types for early embryonic developmental arrest are expectant management, medicinal management with mifepristone and misoprostol and uterine aspiration (Garcia-Grau et al., 2019; Hendriks and MacKenzie, 2019). In recent years, the risk of embryonic arrest in women of childbearing age has increased, leading to widespread concern. Previous studies have reported that early embryonic arrest is related to chromosomal abnormalities, immunity, infection, endocrine dysfunction, environmental physical and chemical factors, among others. However, in 20–40% of cases, the causes are unknown (Fang et al., 2018). In recent years, studies have found that the occurrence of adverse pregnancy outcomes is significantly related to bacterial pathogens and other pathogenic microorganisms in the reproductive tract (Joseph Davey et al., 2016). In particular, the detection of Gardnerella vaginalis in vaginal secretions of patients with bacterial vaginosis (BV) is directly related to embryo arrest. The concentration of *G. vaginalis* in patients with embryo arrest is significantly higher than that in individuals without embryo arrest (Seo et al., 2017; Garcia-Grau et al., 2019).

The vaginal microbiota of women is complex, can change rapidly and dramatically and has a significant impact on women’s health. As we all know, the vaginal secretions of women of normal childbearing age are dominated by *lactobacilli*, which account for more than 95% of the microbial content of the vagina (Antonio and Hillier, 1999). The protective bacteria in the vagina, can inhibit the excessive growth and reproduction of harmful anaerobic and aerobic bacteria, thus maintaining the microecological balance of the vagina. The application of probiotic *lactobacilli* in the clinical treatment and prevention of recurrent reproductive tract infections and vaginal microflora imbalance has become widespread (Chee et al., 2020). Therefore, the abundance and type of *lactobacilli* are important criteria for evaluating the imbalance of vaginal flora (Cohen et al., 2020). Vaginal flora imbalance caused by changes in the vaginal flora is closely related to diseases affecting not only the reproductive health of women during pregnancy but also the embryo. This may be because some infectious pathogens promote the growth of BV-related organisms from the lower genital tract, causing the destruction of the barrier and immune defense mechanism in the inflammatory environment (MacPhee et al., 2010).

With the development of next-generation sequencing (NGS), it has become evident that the endometrium not a sterile environment and that the species diversity and richness of the endometrial community are significantly higher than those of the vaginal environment (Verstraelen et al., 2016; Baker et al., 2018). The uterine microbiomes of women in reproductive age with the high abundance of *Lactobacillus* species, *Lactobacillus iners (L. iners)*, *Prevotella* spp. and *Lactobacillus crispatus (L. crispatus)*, and the endometrial bacteria are characterized by a polymicrobial ecosystem in the pregnant women (Mitchell et al., 2015; Riganelli et al., 2020). Changes to the abovementioned differences in the microbiome suggest that the intrauterine microbiome plays an important role in human health and diseases, and it helps to clarify the pathogenesis of some common gynecological and obstetrical diseases (Toson et al., 2022).

Previous literature has reported the isolation of vaginal microorganisms from the endometrium in 31.4% of women with acute salpingitis and 14.3% of women with pregnancy loss (den Heijer et al., 2019). Bacterial infection can activate the innate immune system, promote the synthesis of nitric oxide and prostaglandins and affect uterine receptivity and embryo implantation. Early embryo arrest not only affects the emotional and mental health of couples seeking to conceive but also causes serious reproductive tract infections. At present, the abundance of Lactobacillus is used to evaluate vaginal flora imbalance (Cohen et al., 2020). Prospective and retrospective cohort studies to assess the impact of vaginal and intrauterine microbiota on embryonic arrest are still lacking. Here, we aimed to identify microbiota differences among women with early embryo arrest and abortion, to improve the microbiota markers related to early embryo arrest.

## Materials and methods

### Study design

Samples were collected from December 2021 to April 2022 in the Obstetrics Clinic of Women’s and Children’s Central Hospital, School of Medicine, University of Electronic Science and Technology of China. 16S rRNA sequencing was performed on vaginal secretions and endometrial samples from 18 pregnant women with normal early pregnancy (N1–N18) and 38 patients with early pregnancy embryonic arrest (T1–T38). Both groups conceived naturally and needed negative pressure suction for artificial termination of pregnancy at 7–10 weeks of pregnancy. The inclusion criteria were as follows: (1) regular menstruation; (2) normal reproductive system development, no comorbidities such as immune diseases, chronic diseases or uterine organic lesions; and (3) no history of exposure to radioactivity or toxic substances, among others; (4) early pregnancy and the presence of a single intrauterine pregnancy sac by ultrasound; (5) exclusion of chromosomal disease in both parents and no family history of genetic conditions; (6) no obvious abnormalities by early laboratory testing, including positive indicators such as mycoplasma, chlamydia, syphilis, AIDS and hepatitis B virus; and (7) no history of sexual activity or vaginal lavage within 3 days before specimen collection, no history of antibiotic medication use in the previous 1 month, and no history of hormone medication in the previous 3 months.

### Sample collection

All participants who met the experimental standards and signed an informed consent form received a gynecological examination conducted by a professional obstetrician. All samples were collected before artificial termination of pregnancy by negative pressure suction. Two sterile cotton swabs were used to collect secretions of the inner wall of the vagina. One of them was tested for the morphology and physicochemical properties of the vaginal secretions, and the other was stored at −80°C for 16S rRNA sequencing. Before the collection of endometrial specimens, a sterile speculum was inserted into the vagina so that the external orifice of the cervix could be seen. To minimize contamination of the vaginal wall and external cervical os with the endometrial microbiome, the cervix was cleaned with gauze soaked in iodine solution prior to endometrial biopsy. According to the literature (Verstraelen et al., 2016), we used an endometrial sampler, which uses a brush covered with plastic on the side and protected by plastic beads at the top to avoid contamination when passing through the vaginal cavity and cervix. After inserting the sheathed brush into the cervical tube, the brush was moved further upward to the uterine cavity, and the brush was pulled out. Then, the brush was rotated five times to collect a specimen from the surface of the entire endometrium. The brush was reshaped before it was removed from the uterine cavity. After the above procedure, the brush was aseptically separated from other parts of the device. Then, endometrial specimens were placed in sterile tubes and frozen at −80°C for 16S rRNA sequencing.

### Vaginal discharge examination

Gram staining was performed on vaginal smears (Dai et al., 2010). After Gram staining, the following assessments were carried out: vaginal cleanliness, white blood cells, Lactobacillus counts, *Candida* spp. spores, blastospores and pseudohyphae, Trichomonas vaginalis counts, clue cell counts, bacterial density, flora diversity and dominant bacteria. Observations followed the National External Quality Assessment System (NSEQA) and College of American Pathologists (CAP) guidelines (Hu et al., 2015).

### 16S rRNA analysis

Sample gDNA purification was performed using the Zymo Research BIOMICS Microprep Kit. Specific primers were used to amplify the V4 region of the sample DNA. The NEW NEBNext UltraII DNA Library Prep Kit for Illumina (ENGLAND BioLabs) was used to build the library. The Illumina HiSeq Rapid SBS Kit v2 was used for high-throughput sequencing. FLASH was used to splice double-ended sequences. QIIME was used to separate each sample sequence from raw reads based on the barcode, and the barcode sequence was cut off. Based on Usearch software,^1^ the UPARSE algorithm was used to cluster operational taxonomic units (OTUs) at a 97% consistency level, and the sequence with the highest frequency in each OTU was selected as the representative sequence of the OTU. To correct for differences in sequencing depth, we randomly subsampled the OTU table to a depth of 29,784 sequences per sample ten times before computing the alpha and beta diversity metrics. UCLUST taxonomy and the SILVA database were used for annotation analysis. Other diagrams were implemented using the R package.

### Statistical analysis

The data were analyzed with SPSS statistical software SPSS 22.0 (IBM, Chicago, IL, United States). The results of normal distribution between groups are expressed as the mean ± standard deviation, and the samples were analyzed by t test. The Kruskal–Wallis test was used for the analysis of microbial differences. *p* < 0.05 indicated that the differences were statistically significant.

### Results

The clinical characteristics of the pregnant women enrolled in this study are shown in Table 1. There was no significant difference in age between the early embryonic arrest and normal pregnancy groups. The gestational age, number of pregnancies and number of miscarriages of the fetuses were also not significantly different between the early embryonic arrest group and the normal pregnancy-induced abortion group (*p* > 0.05).

**Table 1:** General data of the 56 included women.

Characteristics	Early embryonic arrest group (*n* = 38)	Normal pregnancy group (*n* = 18)	Value of *p*
Age (years)	30.68 ± 3.73	30.33 ± 4.74	*0.306*
Gestational age (weeks)	8.00 ± 1.77	7.28 ± 1.36	*0.511*
Number of pregnancies	3.26 ± 1.96	2.56 ± 1.62	*0.278*
Number of miscarriages	2.26 ± 1.93	1.33 ± 1.37	*0.208*

### Comparison of vaginal microecology between the early embryo arrest and normal pregnancy groups

There was no significant difference in vaginal PH, vulvovaginal candidiasis (VVC) or trichomonas vaginitis (TV) between the early embryonic arrest group and the normal pregnancy-induced abortion group. However, the rates of bacterial vaginosis (BV) and vaginal microecological dysbiosis in the early embryonic arrest group were higher than those in the normal pregnancy induced-abortion group (Table 2).

### Dysbiosis of the diversity of the vaginal microbiome in the early embryo arrest group

**Figure 1:**
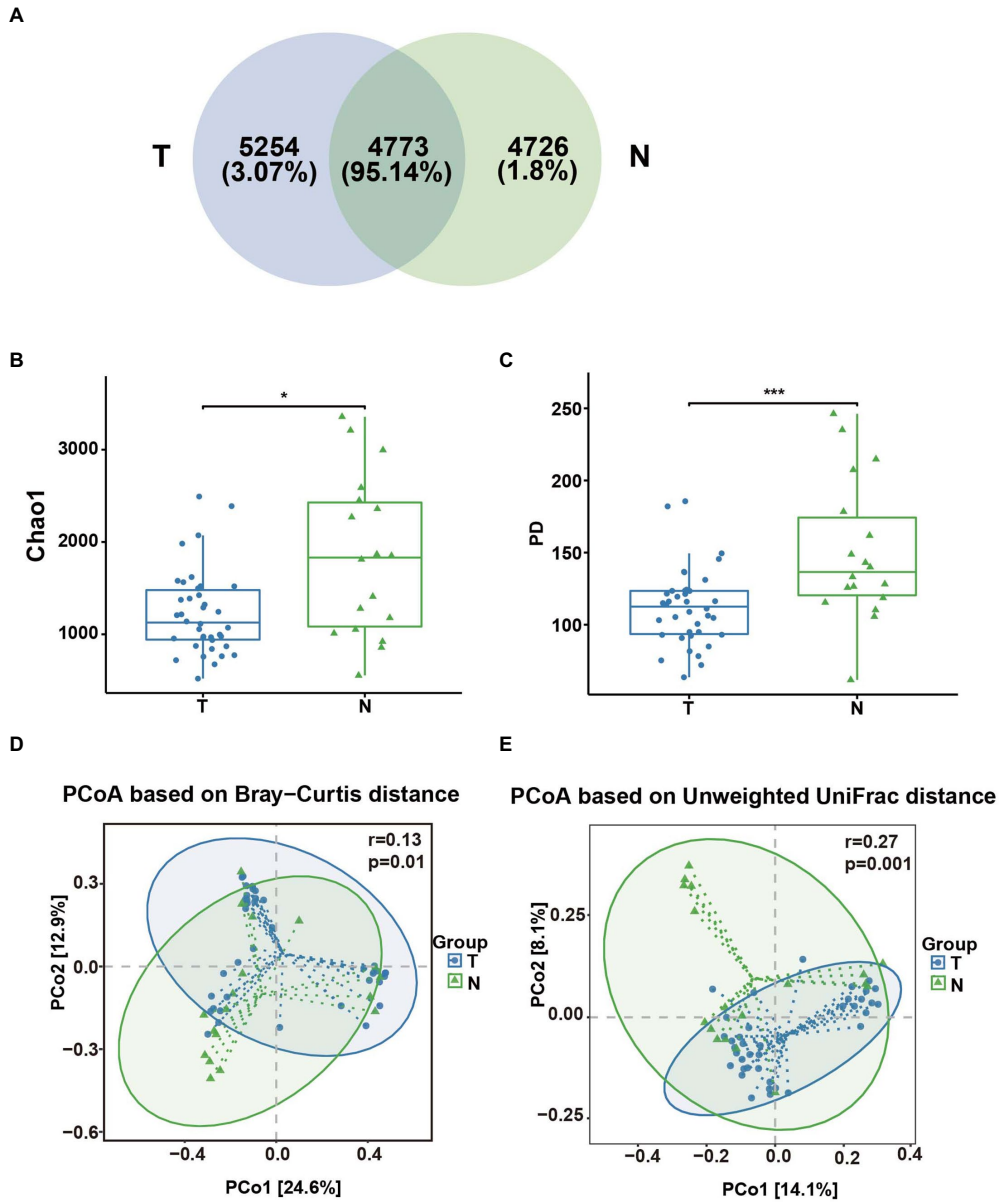
Analysis of the diversity of the vaginal microbial composition in the embryo-arrest and normal pregnancy groups. **(A)** Venn diagram of the numbers of operational taxonomic units (OTUs) identified in the two groups. **(B)** α-diversity of chao1. **(C)** α-diversity of the PD whole tree. **(D)** PCoA plot of the Bray–Curtis distance between the two groups. **(E)** PCoA plot of unweighted Uni-Frac distance between the two groups. T, embryo-arrest group; N, normal pregnancy group. Significance was tested with the Wilcoxon rank-sum test. ^*^p < 0.05; ^***^p < 0.01.

A total of 56 vaginal swab samples were analyzed by 16S rRNA gene sequencing to investigate the relationship between the embryo arrest group and normal pregnancy group. Among the samples, there were 38 vaginal swab samples in the early embryonic arrest group and 18 vaginal swab samples in the normal pregnancy group. A total of 1,921,346 reads were produced by an Illumina MiSeq, with an average of 34,309 reads per sample (shown in Supplementary Table 1). Based on the analysis of rarefaction curves of the two groups, it was found that the rarefaction curves of the two groups of vaginal swabs tended to be flat, indicating that the number of the sequences could cover all the data (shown in Supplementary Figure 1A). A total of 14,753 OTUs were obtained from 56 vaginal swab samples. Vaginal swabs from the early embryonic arrest group were associated with 10,027 OTUs, those from the control group were associated with 9,499 OTUs, and 4,726 OTUs were shared between the two groups (Figure 1A). The Chao1 index and PD whole tree measurements were calculated based on the OTU file to estimate within-sample α-diversity in the vaginal swabs. The Chao1 index (1227.69 ± 453.94 for the T group and 1834.60 ± 861.00 for the N group) and PD whole tree measurements (111.99 ± 26.43 for the T group and 150.04 ± 48.81 for the N group) of the vaginal swabs in the embryonic arrest group were significantly lower than those of the normal pregnancy group (*p* < 0.05; Figures 1B,C). Principal coordinate analysis based on the Bray–Curtis index (*p* = 0.01, *r* = 0.13) and the unweighted UniFrac index (*p* = 0.001, *r* = 0.27) exhibited significant differences in bacterial composition (Figures 1D,E).

### Abundance changes in the vaginal microbiota between the early embryo arrest and normal pregnancy groups

**Figure 2:**
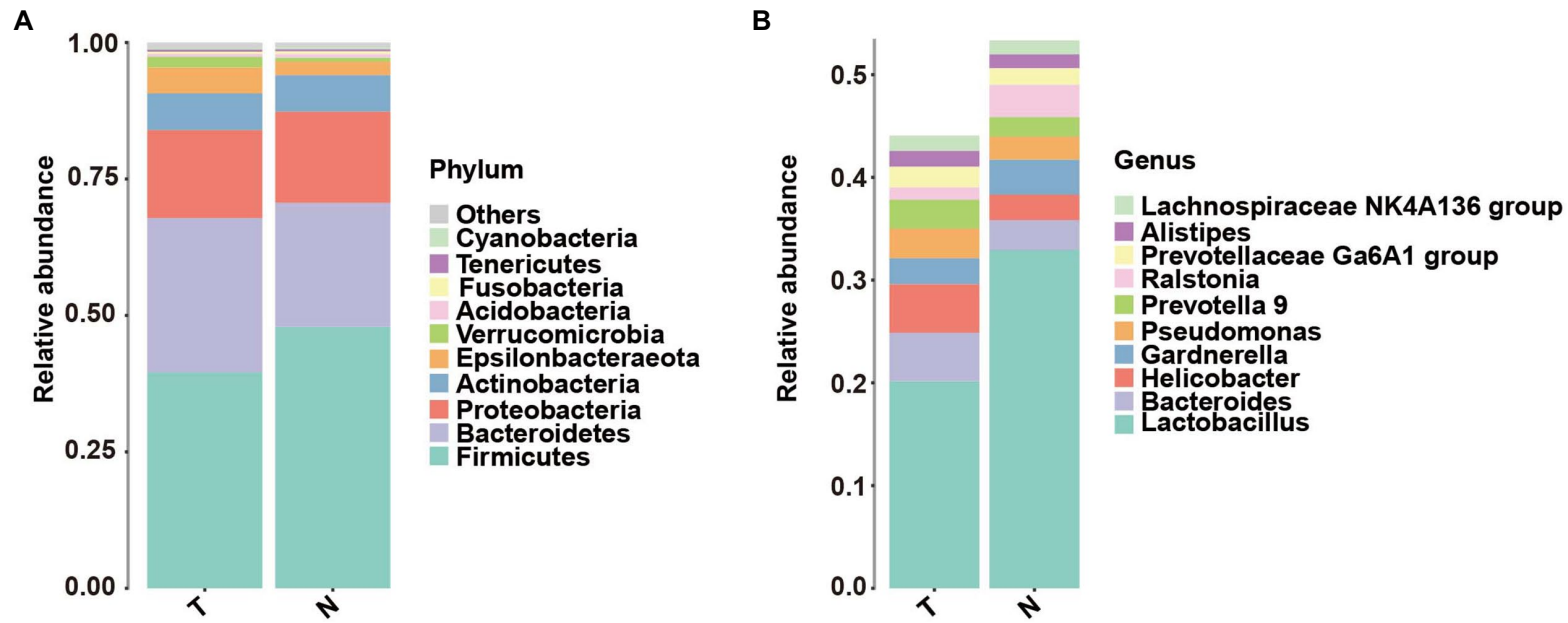
Abundance of microbes at different levels in the embryo-arrest and normal pregnancy groups. **(A)** Relative abundances of the vaginal microbiota at the phylum level. **(B)** Relative abundances of the vaginal microbiota at the genus level. T, embryo-arrest group; N, normal pregnancy group.

The taxonomic classification at the phylum level showed similar patterns in the two groups, which were both dominated by Firmicutes, Bacteroidetes, Proteobacteria and Actinobacteria. However, their relative abundances were not significantly different Figure 2A, (*p* > 0.05). At the genus level, the top 10 genera based on abundance are shown in Figure 2B. The abundance of *lactobacilli* in the normal pregnancy-induced abortion group was higher than that in the embryonic arrest group (*p* < 0.05). Accordingly, the abundance of *Bacteroides* and *Helicobacter* in the early embryonic arrest group was higher than that in the normal pregnancy-induced abortion group (*p* < 0.05). The abundances of *Lactobacillus*, *Gardnerella*, *Helicobacter*, *Prevotella 9*, *Ralstonia*, *Bacteroides* and *Pseudomonas* were higher than 1% in both the normal pregnancy and embryonic arrest groups (Figure 2B), while those of *Megasphaera*, *Pseudarthrobacte*r, *Escherichia–Shigella*, *Sphingomonas*, and *Atopobium* were > 1% only in the normal pregnancy-induced abortion group.

### Discrete microbial communities showing significant differences

**Figure 3:**
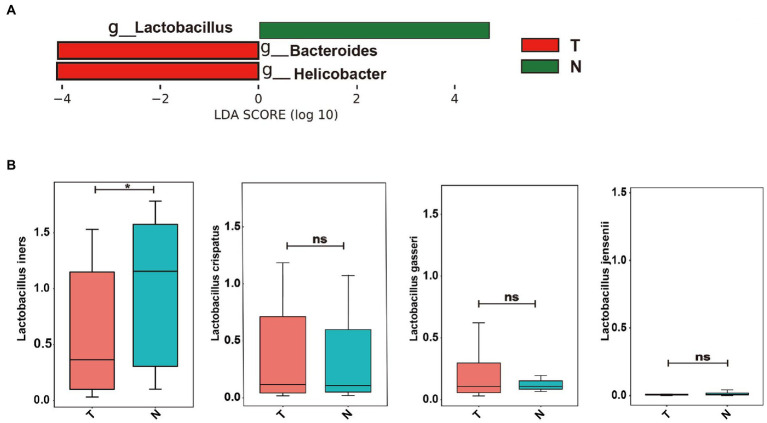
Bacterial taxa analysis of the embryo-arrest and normal pregnancy groups. **(A)** linear discriminant analysis (LDA) scores obtained from the linear discriminant analysis effect size (LEfSe) analysis of the vaginal microbiota in the two groups. **(B)** Relative abundance of *Lactobacillus* is reported with SEM as bar plots. An LDA effect size of > 4 was used as a threshold for the LEfSe analysis. T, embryo-arrest group; N, normal pregnancy group. LDA, linear discriminant analysis; LEfSe, LDA effect size analysis.

To determine whether specific vaginal bacteria were associated with an increased risk of early embryonic arrest, we used linear discriminant analysis effect size (LEfSe) analyses with a linear discriminant analysis (LDA) value of 4.0 at the genus level to identify specific microbial communities in both groups (Figure 3A). LEfSe analysis showed that compared with the normal pregnancy group, the abundance of *Bacteroides* and *Helicobacter* was significantly increased (Figure 3B). The *Lactobacillus* abundance in the normal pregnancy group was significantly higher than that in the embryonic arrest group (Figure 3A). We further analyzed the distribution of Lactobacillus at the species level. *Lactobacillus iners*, *Lactobacillus crispatus*, *Lactobacillus gasseri*, and *Lactobacillus jensenii* were the dominant bacteria (Figure 3B). There were significant differences in *L*. *iners* in the vaginal flora between the embryonic arrest group and the normal pregnancy group (*p* < 0.05). In the embryonic arrest group, the proportion of *L. iners* was significantly decreased (Figure 3B).

### Operational taxonomic unit-based markers on vaginal swabs of embryo arrest

**Figure 4:**
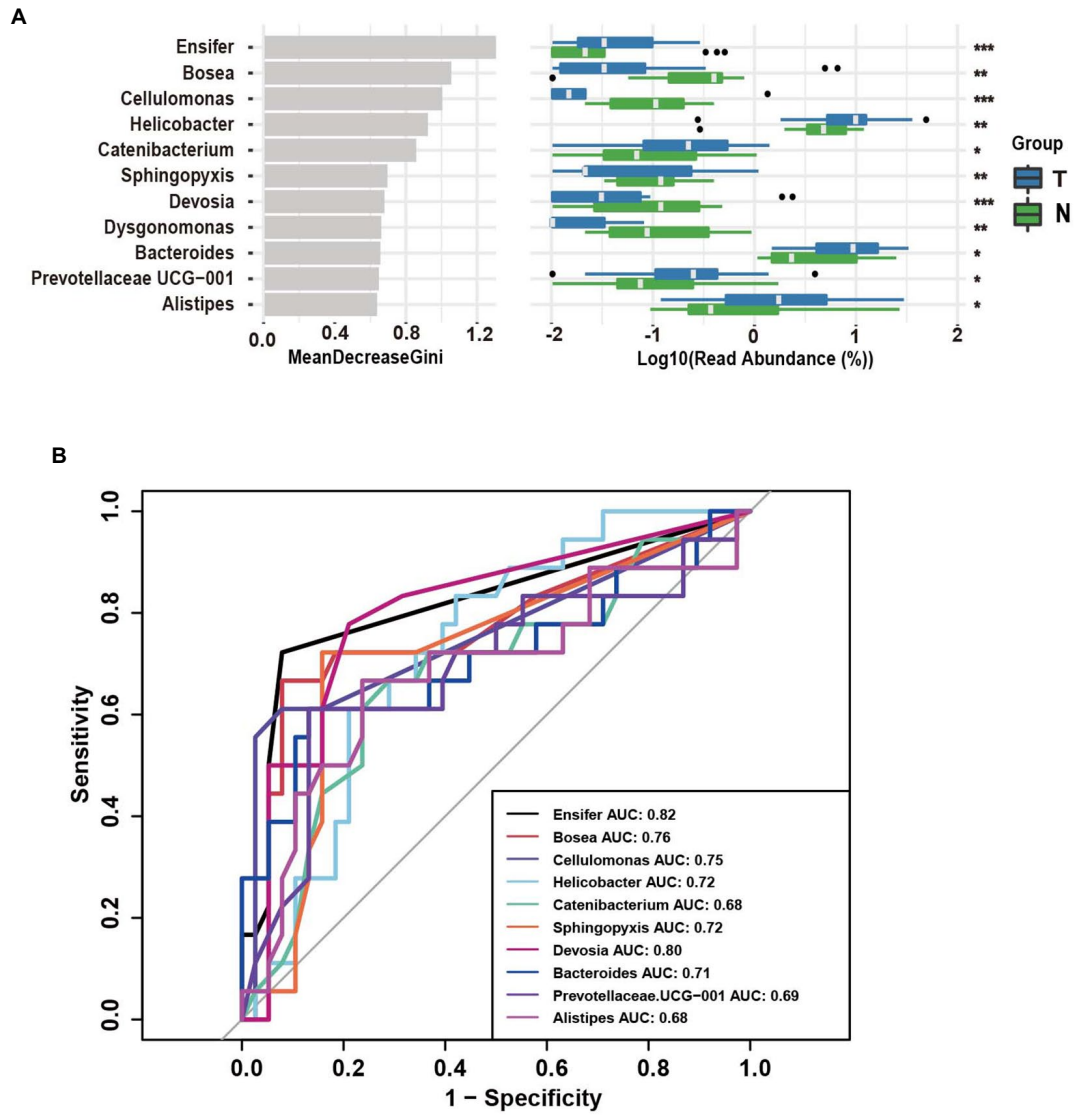
Operational taxonomic unit (OTUs)-based markers on vaginal swabs of the embryo-arrest and normal pregnancy groups. **(A)** Random forest algorithm of the vaginal microbiota in the two groups. **(B)** Receiver operating characteristic (ROC) curve for the prediction of embryo-arrest. T, embryo-arrest group; N, normal pregnancy group.

Based on operational taxonomic units (OTUs), the potential biomarkers of vaginal swabs for the predictive model of embryonic arrest occurrence were examined by the random forest algorithm among the ten different bacterial genera (Figure 4A). The target genera based on the Gini coefficient were predicted by receiver operating characteristic (ROC) curve analysis. In the patients diagnosed with embryo arrest, the area under the curve (AUC) of *Ensifer* and *Devosia* had the highest predictive value. The AUC of *Ensifer* was 0.82, and the AUC of *Devosia* was 0.80. The AUCs of *Bosea*, *Cellulomonas*, *Helicobacter* and *Sphingopyxis* had a certain predictive value (Figure 4B).

### Dysbiosis and taxonomic alterations in the uterine microbiome profiles

**Figure 5:**
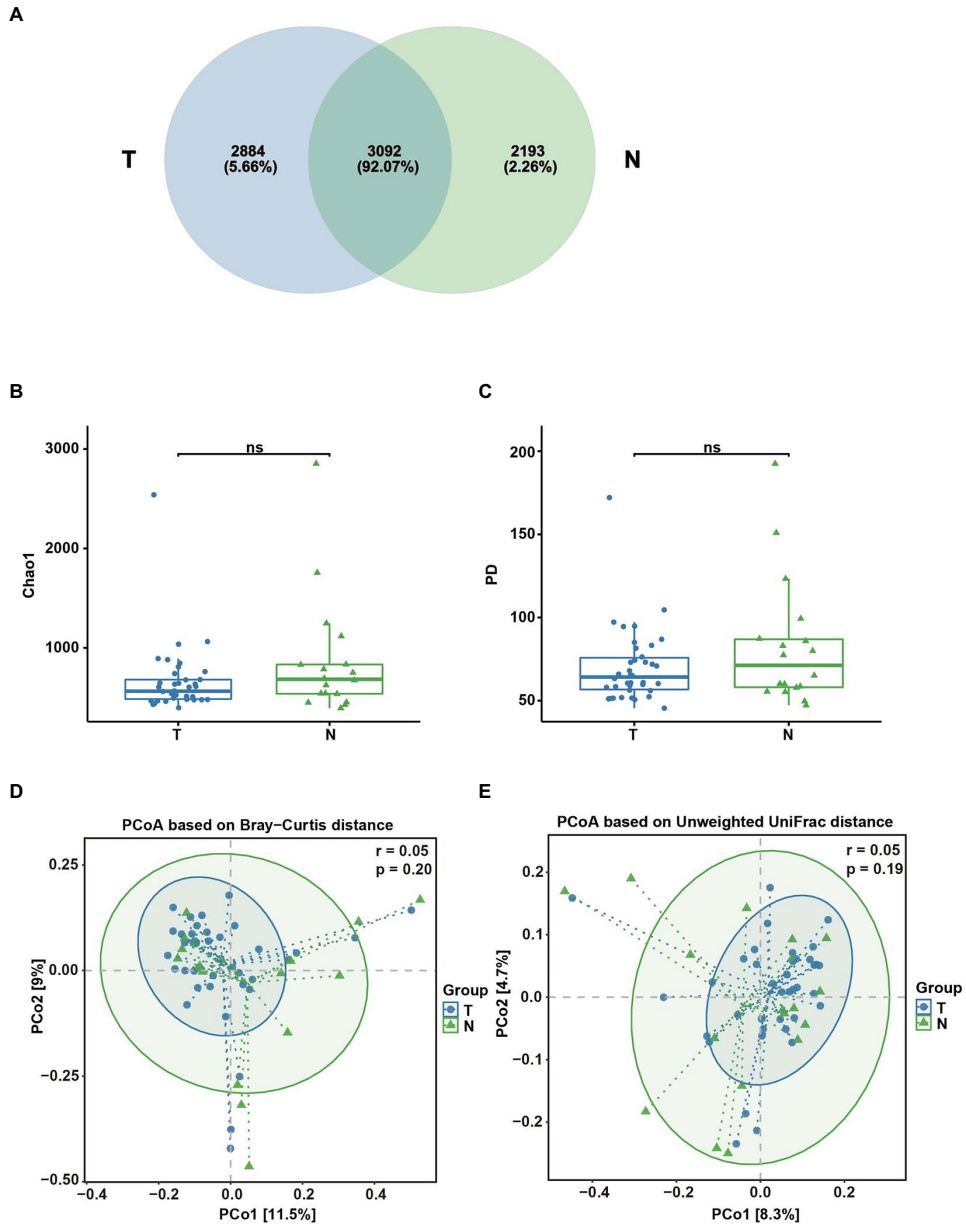
Analysis of the diversity of the uterine microbial composition in the embryo-arrest and normal pregnancy groups. **(A)** Venn diagram of the numbers of OTUs identified in the two groups. **(B)** α-diversity of chao1. **(C)** α-diversity of the PD whole tree. **(D)** PCoA plot of the Bray–Curtis distance between the two groups. **(E)** PCoA plot of unweighted Uni-Frac distance between the two groups. T, embryo-arrest group; N, normal pregnancy group. Significance was tested with the Wilcoxon rank-sum test.

Among the 56 intrauterine tissue samples, there were 38 intrauterine tissue samples in the early embryonic arrest group and 18 intrauterine tissue samples in the normal pregnancy group. A total of 1,920,598 reads were produced by Illumina MiSeq, with an average read count of approximately 34,296 (29,784-38,650; shown in Supplementary Table S1). Based on the analysis of the rarefaction curves of endometrial samples in the embryonic arrest group and the normal pregnancy group, it was found that the curve of the two groups tended to be flat, indicating that the number of sequences was sufficient (shown in Supplementary Figure S1B). There were a total of 8,169 OTUs in the uterine cavity group, 5,976 OTUs in the embryo-arrest group and 5,285 OTUs in the normal pregnancy group, which is similar to 3,082 OTUs, accounting for approximately 92.07% (Figure 5A). Based on the CHAO1 index and PD whole tree measurements, the α-diversity was evaluated, and there was no significant difference between the two groups (*p* > 0.05; Figures 5B,C). Based on the PCoA calculation of the Bray–Curtis distance and unweighted distance, there was no significant difference between the two groups of microbial communities (*p* > 0.05; Figures 5D,E).

### Analysis of bacterial taxa in the uterine endometrium

**Figure 6:**
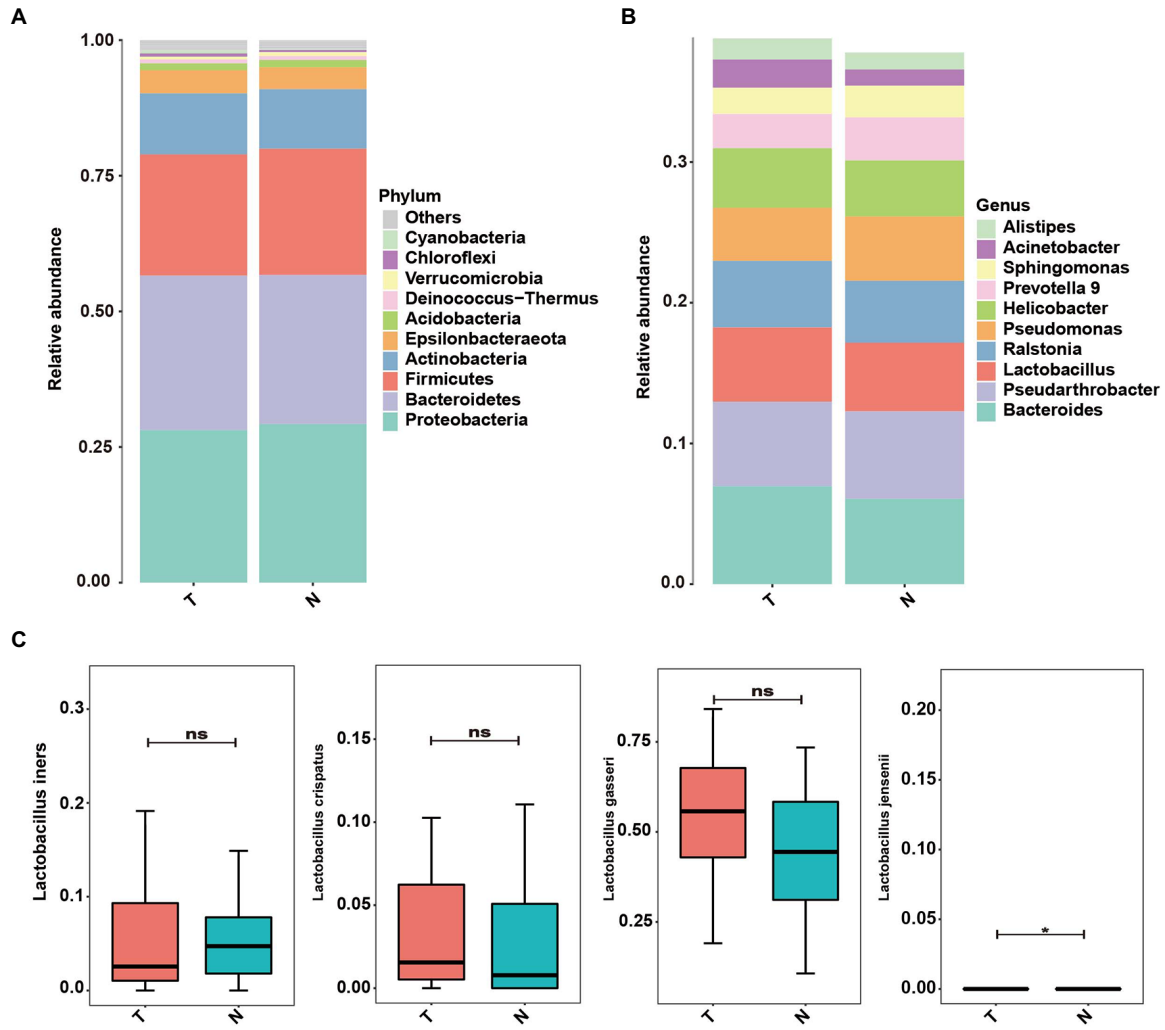
Abundance of microbes at different levels in the uterus of embryo-arrest and normal pregnancy groups. **(A)** Relative abundances of the vaginal microbiota at the phylum level. **(B)** Relative abundances of the vaginal microbiota at the genus level. **(C)** Relative abundance of *Lactobacillus* was reported with SEM as bar plots. T, embryo-arrest group; N, normal pregnancy group.

Proteobacteria, Bacteroidetes, Firmicutes, Actinobacteria and Epsilonbacteraeota were the dominant phyla in the endometrial microbiota in both groups (Figure 6A). At the genus level, *Bacteroides*, *Pseudarthrobacter*, *Lactobacillus*, *Ralstonia*, *Pseudomonas* and *Helicobacter* were the dominant microbial communities in both groups (Figure 6B). In the endometrium, *L. iners*, *L. crispatus*, *L. gasseri*, and *L. jensenii* were the dominant groups of Lactobacillus among the uterine microbial bacteria (Figure 6C). The abundance of *L. jennsenii* was significantly different between the two groups (*p* < 0.05).

### Operational taxonomic unit-based markers of embryo arrest in the vagina

**Figure 7:**
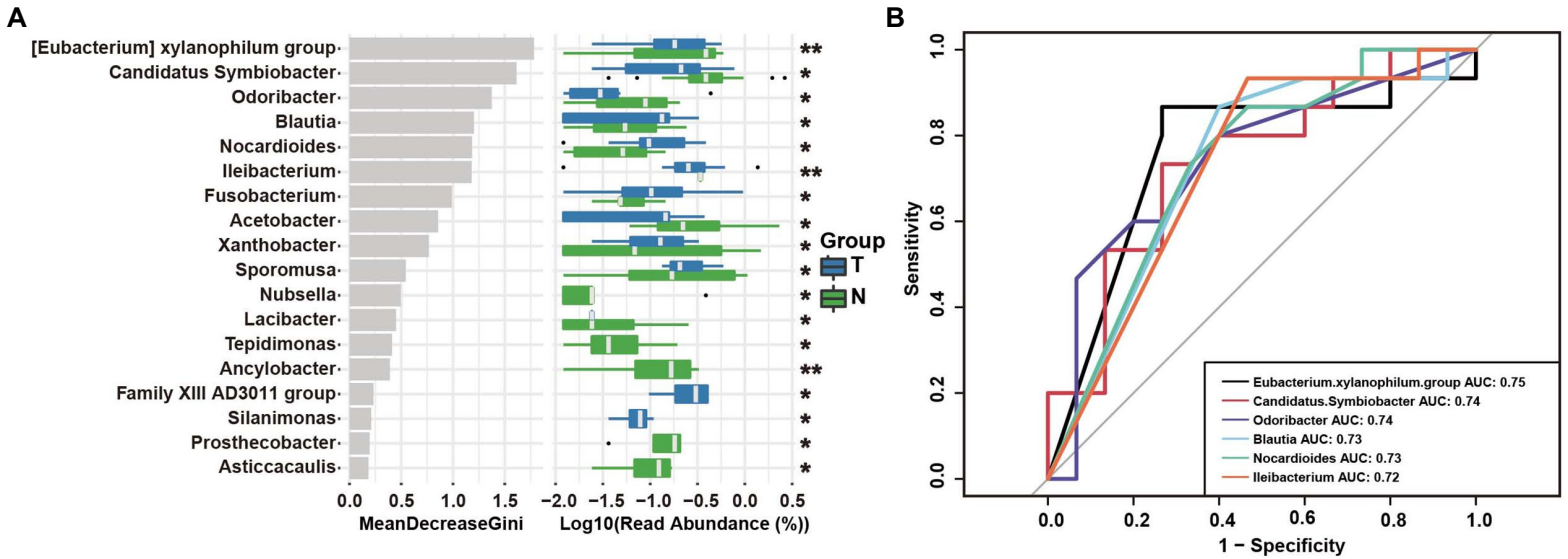
Operational taxonomic unit (OTU)-based markers in the uterus of the embryo-arrest and normal pregnancy groups. **(A)** Random forest algorithm of the uterine microbiota in the two groups. **(B)** Receiver operating characteristics (ROC) curve for the prediction of embryo-arrest. T, embryo-arrest group; N, normal pregnancy group.

The random forest analysis showed 18 genus differences between the two groups (Figure 7A). To evaluate the predictive power of endometrial microbiota for early embryonic arrest, we conducted ROC curve analysis of the six markers with significant differences between the two groups, and the data showed that the prediction value of the *Eubacterium xylanophilum group* was the highest. The AUC was 0.76. The AUCs of *Candidatus Symbiobacter*, *Odoribacter*, *Blautia*, *Nocardioides*, and *Ileibacterium* had a certain predictive value (Figure 7B).

## Discussion

Embryonic arrest refers to the death of the embryo in early pregnancy, with an incidence of 10–15%, and 80% of miscarriages occur within the first 12 weeks of pregnancy (Peters et al., 2020). The incidence of early embryonic arrest has been increasing in recent years, and the high incidence of embryonic arrest has caused serious harm to many women and families (Fang et al., 2018). Therefore, determining the causes of embryonic arrest is the key to preventing the incidence of embryonic arrest from increasing. Previous studies have reported that in women with repeated embryo arrest, the relative abundance of *L. crispatus* in the endometrium was significantly lower than that in healthy women, while the abundance of *Gardnerella vaginalis* in the vagina and endometrium was significantly higher than that in healthy women. Changes in the microecology of the reproductive tract are significantly associated with embryonic development and prognosis (Stout et al., 2017; Peuranpaa et al., 2022). Therefore, the development and prognosis of the reproductive tract microbiome and embryos have attracted increasing attention.

**Table 2:** Analysis of vaginal microecology in the early embryo arrest and normal pregnancy groups.

Microecological factors	Early embryonic arrest group case (%)	Normal pregnancy group case (%)	Value of *p*
Vaginal PH	4.49 ± 0.47	4.31 ± 0.53	*0.491*
BV	14 (36.84)	2 (11.11)	*0.047*
VVC	3 (7.89)	1 (5.55)	*0.751*
TV	0	0	–
Vaginal dysbiosis	24 (63.16)	6 (33.33)	*0.037*

TV, trichomonas vaginitis; VVC, vulvovaginal candidiasis, BV: bacterial vaginosis (BV).

It is known that during pregnancy, due to the dramatic changes in estrogen and progesterone in women of childbearing age, the epithelial cells at the upper end of the vagina grow, and the production of lactic acid in the body increases, which results in changes in the reproductive tract, the pH and natural barriers of the vagina (Chen et al., 2021). The female genital tract accounts for approximately 9% of the human microbiome (Group NHW et al., 2009). Most previous research has focused on the vaginal microbiome, and less research has been conducted on the microbiome in the endometrium. However, dysbiosis of the endometrial microbiota was associated with lower rates of implantation, pregnancy, ongoing pregnancy, and increased spontaneous abortion obstetric complications (Garcia-Grau et al., 2019). The exact relationship between embryonic arrest and the reproductive tract microbiome is still unclear, and there are few correlation studies. Therefore, it is an urgent problem to determine the reason for embryonic arrest to reduce its risk.

Changes in reproductive tract bacteria during pregnancy are characterized by a decrease in the abundance of Lactobacillus and the diversity of the microflora (Di Simone et al., 2020). The most common clinical manifestation of dysbacteriosis during pregnancy is BV of the reproductive tract (Kovachev, 2018; Jayaram et al., 2020). BV is closely related to perinatal maternal and infant health, such as premature rupture of membranes and preterm delivery (Kaambo, 2017; Juliana et al., 2020). In our study, the incidence of BV in the embryo arrest group was significantly higher than that in the normal pregnancy group. In recent years, the clinical incidence of embryo arrest has increased, and some related studies have confirmed that it is closely related to the microflora of the reproductive tract. In the pathogenesis of BV, which is characterized by a decrease in Lactobacillus vaginalis and an increase in Gardnerella, there is no characteristic inflammatory change in the vagina (Yan et al., 2016). Therefore, we further analyzed vaginal swabs for Lactobacillus and Gardnerella. To our surprise, the abundance of Lactobacillus decreased significantly in the embryo-arrest group (shown in Figure 2). However, there was no significant difference in the abundance of Gardnerella. Based on the above research, we speculate that the abundance of Lactobacillus may be more closely related to embryo arrest, which is also consistent with previous studies (Al-Memar et al., 2019).

The dominant genus Lactobacillus in the vagina of women of childbearing age is a protective bacterium of the vaginal microbiome (Kalia et al., 2020). In patients undergoing *in vitro* fertilization (IVF), the success rate of IVF is higher if only Lactobacillus is present in the vagina at different time points. Lactobacillus may play a certain role in pregnancy, embryo colonization and normal development during pregnancy (Leitich and Kiss, 2007; Hyman et al., 2012). Previous studies have also confirmed that the bacteria in the female vagina are dominated mainly by lactobacillus species, namely, *L. crispatus*, *L. iners*, *L. jensenii*, and *L. gasseri* (Romero et al., 2014; DiGiulio et al., 2015; Witkin et al., 2019, 2021). The vaginal microbiome of healthy women has been classified into five community state types (CSTs) according to their community structure. Four types of CST are dominated by Lactobacillus, namely *L. crispatus* (CST I), *L. gasseri* (CST II), *L. Iners* (CST III) and *L. jenesii* (CST V). CST IV is characterized by low levels or absence of *Lactobacillus* (Ravel et al., 2011; Liu et al., 2021). Previous studies have reported that *lactobacilli* in the vagina during pregnancy are dominated mainly by *L. crispatus* (CST I). In addition, the higher the abundance of *L. crispatus* is, the lower the abundance of other *Lactobacillus* species will be (Witkin et al., 2021). To explore the role of the community state types (CSTs) of Lactobacillus in embryonic development, we further classified *Lactobacillus* in the vagina. In our study, both groups were dominated by *L. iners* (CST III), but the abundance of *L. iners* in the embryo-arrest group was significantly lower than that in the normal pregnancy group (shown in Figure 3). At present, research on *L. iners* is still controversial, and its exact function in the vagina is uncertain. Studies have confirmed that *L. iners* is a beneficial species and is related to vaginal dynamic balance. It can survive and help restore vaginal homeostasis (Ferris et al., 2004; Petrova et al., 2017). In the transitional stage of the vaginal microbiota, it may become the dominant species related to term delivery in pregnant women and associated with a high risk of adverse pregnancy outcomes (Jakobsson and Forsum, 2007; Romero et al., 2014; Stout et al., 2017). Our research shows that the balance of vaginal microbiology may play a certain role in the occurrence of embryo arrest and that domination of the internal environment by different lactobacilli would have different effects vaginal health in women.

At present, the study of the vaginal microbiome is still the focus of research, and an increasing number of studies have focused on changes in the vaginal microbiome during pregnancy, to infer its impact on pregnancy outcomes. Previous studies have confirmed that the abundance of Lactobacillus increases and that the diversity of bacteria decreases during pregnancy. In our study, the diversity of bacteria in the embryo arrest group decreased significantly, and Firmicutes, Bacteroidetes, Proteobacteria and Actinobacteria were dominant in the two groups at the phylum level; however, at the genus level, the abundance of Lactobacillus in the embryo arrest group decreased significantly, and the corresponding Bacteroides and Helicobacter increased significantly. To identify the bacteria related to embryo arrest in the vagina of the embryo arrest group and the normal pregnancy group, we used random forest analysis to identify the different bacteria and further predict embryo arrest by ROC curve analysis. We found that *Ensifer* and *Bosea* had high values in embryo arrest. In this study, we detected two special strains, Ensifer and Bosea. Ensifer was isolated in 1982 and located in the soil. Previously, scholars have reported that Ensifer microbes may be temporary in the skin, and have been found to be present in human skin for hours to days (Suwarsa et al., 2021). Bosea was detected in cervical microflora in a study of the relationship between human papillomavirus infection and age, and it was significantly associated with HPV infection (Hu et al., 2022). Interestingly, these two strains were detected in the vagina of pregnant women with embryo arrest and normal pregnant women, their abundances were low, and there was a significant difference between the two groups; however, their specific effects need to be further studied. The development of next-generation sequencing techniques has improved our understanding of the vaginal microbiota, which represents a unique microbiota (Franasiak et al., 2016; Verstraelen et al., 2016).

For a long time, research on the microbiota of the female reproductive tract during pregnancy has focused mainly on the vagina. Due to the limited collection of intrauterine samples during pregnancy, there are few studies on intrauterine flora during pregnancy (Moreno and Simon, 2018; Riganelli et al., 2020). Previous studies have shown that bacteria can enter and reproduce in the uterus from the vagina and cervix (Ramos Bde et al., 2015). The changes in the endometrial microbiome, characterized by a low abundance of lactobacilli, seem to be related to the maintenance and implantation of embryos. However, the current reports of the microbiota in the uterus vary widely. Due to the low abundance of the microbiome in the uterus, fewer changes in the vagina and uterus, and the possibility of sample contamination is inevitable (Baker et al., 2018). The new study is still exploring the interaction between the microbial profiles in the uterine cavity and the host during pregnancy. At present, the study of the uterine microbiome still needs to overcome some technical challenges to avoid misleading conclusions. The abortion caused by the stagnation of early embryonic development usually occurs in the early stage of pregnancy, so the changes in microflora in the uterine cavity of pregnant women have an important impact on the changes in embryos. We further attempted to use high-throughput sequencing to discover the unique microbiota in the uterine cavity of patients with embryonic arrest and women during normal pregnancy. The bacterial diversity of in the uterine cavity was not significantly different between the embryo-arrest group and normal pregnancy group. The core assemblage microbiota at the phylum level were Proteobacteria, Bacteroidetes, Firmicutes and Actinobacteria similar to the vaginal microbiota. However, the abundances were significantly different from that of the vaginal microbiome. The microbiota in the uterus has the highest proportion of Proteobacteria, and the highest proportion of Firmicutes in the vagina (Toson et al., 2022). At the genus level, we observed that *Bacteroides*, *Pseudarthrobacter*, *Lactobacillus*, *Ralstonia* and *Pseudomonas* accounted for the main proportion between the embryo-arrest group and the normal pregnancy group. This is consistent with previous studies reporting that non-*Lactobacillus* was the dominant genus (Mei et al., 2019; Moreno et al., 2020). However, it has also confirmed that the microbiome in the uterus is not dominated by lactobacilli (Leoni et al., 2019). The bacteria in the endometrium mentioned above not only include Lactobacillus, but are also significantly related to pregnancy.

There is a certain relationship between the imbalance in endometrial microbiota and the determination of the cause of infertility, and changes in the microbiome of may be the reason for disease in women (van Oostrum et al., 2013; Moreno and Simon, 2018). The cervix forms a complete barrier between the uterus and vagina. The mucins in the cervix change their conformation, allowing bacteria to pass through under certain conditions (Brunelli et al., 2007). It has been confirmed that there is a significant correlation between the abundance of lactobacilli and pregnancy outcomes in recurrent pregnancy loss. The abundance of Lactobacillus was significantly correlated with implantation, pregnancy and live birth rate in women. The survival rate of embryos is higher when the microbiome of the uterine cavity comprises Lactobacillus-dominant microbiota at the time of implantation, and pregnancy (Moreno et al., 2016; Shi et al., 2022). However, infection of the pathogenic microbiome in the reproductive tract, especially inflammatory reactions, destroys the structure of the chorion and amniotic membrane, which is toxic to the endometrium and the normal development of embryos, making this an important cause of embryo arrest (Giakoumelou et al., 2016). In our study, we explored the relationship between the endometrial microbiome and pregnancy outcomes and observed significant changes in the intrauterine cavity microbiome in the embryo-arrest group and the normal pregnancy group. We further classified the Lactobacillus, and found that the Lactobacillus in the uterus is also dominated by *L. iners*, *L. crispatus*, *L. gasseri* and *L. jensenii*. To our surprise, *L. jensenii in utero* was significantly different between the embryo-arrest group and normal pregnancy groups. In the vagina, the abundance of the above microbiota is significantly related to preterm delivery. In pregnant women with missed abortion, this abundance is significantly decreased (Sun et al., 2022). The exact role of Lactobacillus species in the uterine cavity has not been reported thus far and its exact effect on embryo arrest remains to be explored. More research is still needed on the relationship between the microflora in the uterine cavity and embryo arrest in order to characterize the different microbiomes *in utero* and evaluate their predictive value for embryo arrest. Through random forest analysis, we analyzed the ROC curves of the bacteria with significant differences in the uterus. We found that the *Eubacterium xylanophilum group*, Candidatus Symbiobacter, Odoribacter, Blautia, *Nocardioies* and *Ileibacterium* have a certain predictive value for embryo arrest. Thus, the specific mechanism of the above bacteria in the implantation and outcome of embryos in the uterus as well as the interactions of bacteria in the uterine cavity need to be further studied.

Thus, it can be seen that the balance of vaginal microecology and uterine microflora during pregnancy is very important for the development of embryos. There are many reports on the balance of microecology in the vagina during pregnancy, but it is also affected by a variety of factors, including the interaction of bacteria to maintain the relative balance of the vagina. Embryo arrest is a complex process, not only in the vaginal environment, but also in the uterine cavity. Our study explored the effect of the environment in the reproductive tract on embryo arrest from two aspects of the difference in vaginal and uterine flora, to provide some research ideas for the prevention of embryo termination.

## Conclusion

With the support of high-throughput sequencing technology, we performed a detailed study on the uterine and vaginal microbiomes of patients with embryo arrest and women with normal pregnancies. There were significant differences in the uterine and vaginal microbiomes between these two groups. The above study of the microbiome provides insights into the pathogenesis of embryo arrest and, according to our evidence. Additionally, our findings indicate that some bacteria have a certain predictive value for embryo arrest. However, due to the limitation of sample size and specimen collection, the predictive value of the microbiome in the uterus and vagina for embryo termination is limited, and further prospective studies are needed to determine the predictive value of the microbiome.

## Data availability statement

The data presented in the study are deposited in the NCBI repository, accession number PRJNA933192.

## Ethics statement

The studies involving human participants were reviewed and approved by Chengdu Women’s and Children’s Central Hospital (grant no. 2021(88)). The patients/participants provided their written informed consent to participate in this study.

## Author contributions

LW and ML contributed to the study design. LH, JC, HLiu, and YL completed all tests. LW, ML and WL analyzed the data and drafted this paper. HLi and ZL contributed to reagent management. All authors contributed to the article and approved the submitted version.

## Funding

The clinical case screening and data collection were supported by the Chengdu Science and Technology Bureau: technology innovation research and development project [2021-YF05-00648-SN] and Chengdu Medical Research Project [2022203].

## Conflict of interest

The authors declare that the research was conducted in the absence of any commercial or financial relationships that could be construed as a potential conflict of interest.

## Publisher’s note

All claims expressed in this article are solely those of the authors and do not necessarily represent those of their affiliated organizations, or those of the publisher, the editors and the reviewers. Any product that may be evaluated in this article, or claim that may be made by its manufacturer, is not guaranteed or endorsed by the publisher.

## Footnotes

^1^ http://drive.com/uparse/
