# Supplementary figures and images for: Association between the vaginal and uterine microbiota and the risk of early embryonic arrest
Source: Front Microbiol. 2023 Mar 22;14:1137869. doi: 10.3389/fmicb.2023.1137869 (PMC10073571; doi:10.3389/fmicb.2023.1137869)

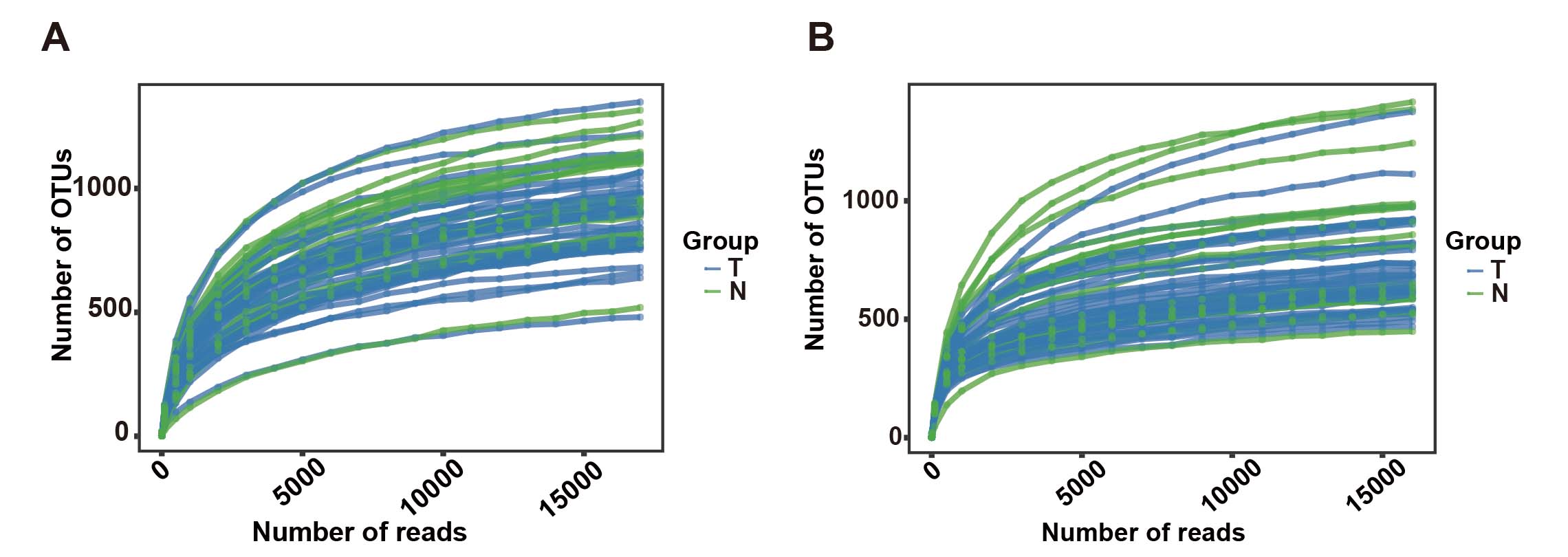

Supplement: Supplementary file 1 [file Image_1.JPEG]
